# Co-ordination of NDH and Cup proteins in CO_2_ uptake in cyanobacterium *Synechocystis* sp. PCC 6803

**DOI:** 10.1093/jxb/erx129

**Published:** 2017-05-10

**Authors:** Xunling Han, Nan Sun, Min Xu, Hualing Mi

**Affiliations:** 1 National Key Laboratory of Plant Molecular Genetics, Institute of Plant Physiology and Ecology, Shanghai Institutes for Biological Sciences, Shanghai, China; 2 University of Chinese Academy of Sciences, Beijing, China

**Keywords:** CO_2_ uptake, CupA, CupB, NDH-1MS, NDH-1MS′, *Synechocystis* sp. PCC 6803

## Abstract

High and low affinity CO_2_-uptake systems containing CupA (NDH-1MS) and CupB (NDH-1MS′), respectively, have been identified in *Synechocystis* sp. PCC 6803, but it is yet unknown how the complexes function in CO_2_ uptake. In this work, we found that deletion of *cupB* significantly lowered the growth of cells, and deletion of both *cupA* and *cupB* seriously suppressed the growth below pH 7.0 even under 3% CO_2_. The rate of photosynthetic oxygen evolution was decreased slightly by deletion of *cupA* but significantly by deletion of *cupB* and more severely by deletion of both *cupA* and *cupB*, especially in response to changed pH conditions under 3% CO_2_. Furthermore, we found that assembly of CupB into NDH-1MS′ was dependent on NdhD4 and NdhF4. NDH-1MS′ was not affected in the NDH-1MS-degradation mutant and NDH-1MS was not affected in the NDH-1MS′-degradation mutants, indicating the existence of independent CO_2_-uptake systems under high CO_2_ conditions. The light-induced proton gradient across thylakoid membranes was significantly inhibited in *ndhD*-deletion mutants, suggesting that NdhDs functions in proton pumping. The carbonic anhydrase activity was suppressed partly in the *cupA*- or *cupB*-deletion mutant but severely in the mutant with both *cupA* and *cupB* deletion, indicating that CupA and CupB function in conversion of CO_2_ to HCO_3_^–^. In turn, deletion of *cup* genes lowered the transthylakoid membrane proton gradient and deletion of *ndhD*s decreased the CO_2_ hydration. Our results suggest that NDH-1M provides an alkaline region to activate Cup proteins involved in CO_2_ uptake.

## Teaser

NdhD4 and NdhF4 are essential for the assembly of CupB-containing complexes. Both NDH and Cup proteins are involved in CO_2_-uptake systems and co-ordinate CO_2_ uptake activity.

## Introduction

Cyanobacteria possess a CO_2_-concentrating mechanism (CCM) that enables the accumulation of inorganic carbon (HCO_3_^–^ and CO_2_, collectively called Ci) at the carboxylation site to a level for efficient CO_2_ fixation despite the low affinity of their Rubisco for CO_2_ (Kaplan and Reinhold, 1999; Ogawa and Kaplan, 2003). The CCM requires the coordination of two systems, an inorganic carbon transporter system and the carboxysome containing Rubisco. To date, five inorganic carbon transporters have been found, including two Na^+^-dependent HCO_3_^–^ transporters (BicA and SbtA), one ATPase-dependent HCO_3_^–^ transporter (BCT1), and two CO_2_-uptake NDH-1 complexes, in *Synechocystis* sp. PCC 6803 (hereafter *Synechocystis* 6803) and other cyanobacterial strains (Ogawa and Kaplan, 2003; Ogawa and Mi, 2007; Price, 2011). One of the CO_2_ uptake complexes, the NDH-1MS′ complex, consists of NdhD4, NdhF4 and CupB (ChpX) and is a constitutive system with weaker uptake affinity for CO_2_; another one, the NDH-1MS complex, consists of NdhD3, NdhF3 and CupA (ChpY), and is inducible at limiting Ci conditions and has a higher uptake affinity for CO_2_ (Ohkawa *et al.*, 2000*b*; Shibata *et al.*, 2001; Maeda *et al.*, 2002). According to the mutant phenotype, the expression of the *ndhF3-ndhD3-cupA-sll1735* operon is induced in both *Synechocystis* 6803 and *Synechococcus* sp. PCC 7002 cells grown under low CO_2_ condition (Ohkawa *et al.*, 2000*b*; Shibata *et al.*, 2001; Maeda *et al.*, 2002). Further research showed that the proteins encoded by *ndhF3-ndhD3-cupA-sll1735* form a small complex, NDH-1S, in which CupA and a small protein, CupS, were identified as subunits by proteomic analysis (Zhang *et al.*, 2004, 2005). The *ndhB*-defective mutant M55 was shown to be unable to grow under low CO_2_ conditions even when NDH-1S was present, suggesting that the normal operation of the CO_2_-uptake system requires both NDH-1M and NDH-1S. A complex (NDH-1MS) containing both NDH-1S and NDH-1M has been isolated from a *Thermosynechococcus elongatus* strain in which the C-terminus of NdhL has been tagged with 6xHis. This complex is easily dissociated into NDH-1M and NDH-1S complexes (Zhang *et al.*, 2005; Battchikova *et al.*, 2011). NDH-1MS has been characterized as a U-shape structure by analysis by single particle electron microscopy after purification from the thylakoid membranes of *Thermosynechoccus elongates* (Arteni *et al.*, 2006). CupA is responsible for the U-shape by binding at the tip of the membrane-bound arm of NDH-1MS in *Thermosynechoccus elongatus* and *Synechocystis* 6803 (Folea *et al.*, 2008). Although the constitution and the function of NDH-1MS have been studied, the underlying mechanism explaining the functional link between NDH-1M and NDH-1S or NDH-1S′ still remains to be resolved.

Reverse genetic studies have indicated an essential role for the *cupB* gene, a homolog of *cupA*, in constitutive CO_2_ uptake and suggest that the proteins encoded by *ndhF4-ndhD4* and *cupB* form the small complex, NDH-1S′ (Shibata *et al.*, 2001; Maeda *et al.*, 2002). In a previous study, we reported that the CupB protein in *Synechocystis* resides in the thylakoid membrane but is missing from the *ndhD4* deletion mutant (Xu *et al.*, 2008). Based on the purification of a 450 kDa complex containing both NdhH and CupB proteins, we suggested that the complex is NDH-1MS′ residing in the thylakoid membranes. However, the function of NDH-1MS′ still needs elucidation.

It has been suggested that there exist two independent CO_2_ uptake systems, NDH-1MS and NDH-1MS′, in which NdhDs function in proton pumping (Battchikova *et al.*, 2011), Cups function in CO_2_ hydration as carbonic anhydrase (CA)-like proteins (Kaplan and Reinhold, 1999; Price, 2011). However, whether NDH-1MS induced by low CO_2_ exists or functions under high CO_2_ conditions, and whether or how it relates to the constitutive type NDH-1MS′ still remain to be clarified.

In this work, we investigated the function of NDH-1MS and NDH-1MS′ in CO_2_ uptake using reverse genetics and biochemical methods. Our results suggest that NDH-1MS and NDH-1MS′ are essential for efficient CO_2_ uptake especially under changed pH conditions. We proposed a model for the function of CO_2_ uptake systems in *Synechocystis* 6803.

## Materials and methods

### Cell culture conditions

Wild type（WT）and mutant cells of *Synechocystis* 6803 were grown at 30 °C in 50 ml liquid BG11 medium buffered with 5 mM Tris-HCl (pH 8.0) and bubbled with 3% v/v CO_2_ in air at 3 ml min^–1^. The mutant strains were grown in liquid BG11 medium with appropriate antibiotics, and cell cultures were harvested at the logarithmic phase (OD_730_=0.6–0.8). Solid medium was BG11 supplemented with 1.5% agar. Continuous illumination was provided by fluorescent lamps, generating 50 μmol of photons m^–2^ s^–1^.

### Construction and isolation of mutants

Construction of single mutants, such as *ΔcupA*, *ΔcupB*, *ΔndhD1*, *ΔndhD2*, *ΔndhD3*, *ΔndhD4*, *ΔndhL*, *ΔndhK*, and M55, has been described in previous studies (Ogawa, 1991; Ohkawa *et al.*, 2000*a*; Shibata *et al.*, 2001). BHM is a *Synechocystis* 6803 mutant with CupB tagged with 6xHis-cMyc at the C-terminus, which has also been described before (Xu *et al.*, 2008). Those constructions of single mutants were used to transform various appropriate mutants to generate the double mutants or triple mutants, i.e. *ΔcupA/B*, *ΔndhD1/D2*, *ΔndhD3/D4*, and *ΔndhD1/D2/D3*. The *ΔndhF4*/BHM and *ndhL-YFP-6His* were also described in previous studies (Xu *et al.*, 2008; Birungi *et al.*, 2010). To construct the *ΔndhF1* mutant, its coding region was inserted by a chloramphenicol (CM) resistance cassette as follows. The *ndhF1* upstream and downstream regions were amplified using primer pairs hxl108/hxl109 and hxl112/113, with CM resistance cassette using primers hxl110/hxl111 (see Supplementary Table S1 at *JXB* online). These three fragments were used as the template to synthesize an Up-CM-Dn fragment through overlap PCR, and this was ligated into the T-vector to make the construct for transforming the wild type of *Synechocystis* 6803 (Supplementary Fig. S1A). The *ΔndhF3* mutant was made with the same strategy, using primer pairs hxl114/hxl115 and hxl118/119, with the CM resistance cassette using the primers pair hxl116/hxl117 (see Supplementary Table S1). The plasmids were separately transformed into BHM to generate the double mutants, *ΔndhF1*/BHM and *ΔndhF3*/BHM. The mutated genes in the transformants were segregated to homogeneity (by successive streak purification) as determined by PCR amplification (see Supplementary Fig. S1B).

### Isolation of soluble fractions and total membrane fractions

Soluble fractions and total membrane fractions of *Synechocystis* 6803 cells were isolated as described previously with slight modifications (He and Mi, 2016).

### Electrophoresis and immunoblotting

SDS-PAGE of thylakoid membranes from *Synechocystis* 6803 was carried out on a 1.0 mm thick, 12% polyacrylamide gel (Laemmli, 1970). Blue native (BN)-PAGE of *Synechocystis* 6803 membranes was performed as described previously with modifications from He *et al.* (2016). After electrophoresis, the proteins were electrotransferred to polyvinylidene difluoride (PVDF) membranes and detected with specific antibodies. Finally, an ECL assay kit was used according to the manufacturer’s protocol. The CupB antibody was prepared against 156 amino acids of the C-terminus, which was expressed in a pET-51b(+) vector and isolated with a His tag in our lab (see Supplementary Fig. S2); it was made by the Shanghai Immune Biotech Co. Ltd (China). Antibodies against NdhA, NdhB, NdhK, and YFP were raised in our laboratory and were used in previously published work (Hu *et al.*, 2013).

### CO_2_ uptake measurements

Wild type and mutants of *Synechocystis* 6803 were grown in BG11 medium bubbled with 3% v/v CO_2_ in air until logarithmic phase. Cell cultures were harvested by centrifugation at 5000 *g* for 10 min and suspended in BG11 buffer, pH 8.0, to a final concentration of OD_730nm_ of 100. Then, 30 µl of the cells were spotted on agar plates containing BG11 buffer at pH 8.0. A slice (1 cm×1 cm) of solid BG11 medium containing the cells was cut off and put on a piece of microscope coverglass. After that, CO_2_ uptake was measured by an Li-6400 XT portable photosystem with the concentration of CO_2_ controlled at 2%, 1% or 0.04% (v/v in air) as described previously (Chen *et al.*, 2016). Three independent measurements were performed and CO_2_ uptake activities were calculated from concentration of chlorophyll. Light intensity and temperate were 100 µmol photons m^–2^ s^–1^ and 30 °C, respectively.

### Quinacridine fluorescence quenching

Fluorescence of quinacridine (QA) at 503 nm was measured using the PAM chlorophyll fluorometer (Maxi-version, Walz, Effeltrich, Germany) attached to a US-370 emitter with an emission peak at 375 nm and a PM-101/D detector as described previously (Xu *et al.*, 2014; Chen *et al.*, 2016). Cells were harvested at logarithmic phase and suspended in reaction mixture of fresh BG11 medium with 5 μM QA at a final chlorophyll concentration of 10 μg ml^–1^. The quenching of QA fluorescence was induced by illuminating the cells with actinic light (60 μmol photons m^–2^ s^–1^) after the background fluorescence became stable after about 2 min.

### Carbonic anhydrase activity measurement

The carbonic anhydrase assay was based on the rate of change in pH value after the injection of a standard amount of CO_2_-saturated water, as described previously with modification (Jiang *et al.*, 2013). The cell cultures (1 l) were harvested at the logarithmic phase and were broken by vortexing five times at the highest speed for 20 s at 4 °C with a beadbeater (Biospec) followed by 3 min cooling on ice. Then the thylakoid membranes were separated and suspended in assay buffer (20 mM sodium barbital, pH 8.2, 10 mM MgCl_2_ and 0.5 mM phenylmethylsulfonyl fluoride) at a final chlorophyll concentration of 1 mg ml^–1^. Then the sample corresponding to 100 μg chlorophyll was added to 8 ml 20 mM sodium barbital, pH 8.2 with a pH electrode inserted into the assay solution. After the temperature equilibrated at 4 °C, 4 ml ice-cold CO_2_-saturated water was injected, and then the time for the pH to change from 8.0 to 7.0 was recorded. The buffer without membrane fraction was used as the control. CA activity was calculated as the difference in the initial rate of CO_2_ hydration between the control and the samples. CA activity is expressed in Wilbur–Anderson units (WAU) per mg of chlorophyll. One WAU is defined as 10×(*t*_0_–*t*)/*t*, where *t*_0_ and *t* are the times required for the pH change in the control and the sample, respectively (Jiang *et al.*, 2013).

### Oxygen exchange

The rate of O_2_ evolution in the cells of wild type and mutants was determined using a Clark-type O_2_ electrode at 30 °C as described previously (He and Mi, 2016). The cell at logarithmic phase was used for the measurement in the presence of 10 μM NaHCO_3_ and the light intensity used to induce the photosynthetic oxygen evolution was 1000 μmol photons m^–2^ s^–1^.

## Results

### Growth and CO_2_-uptake characteristics

**Fig. 1.**
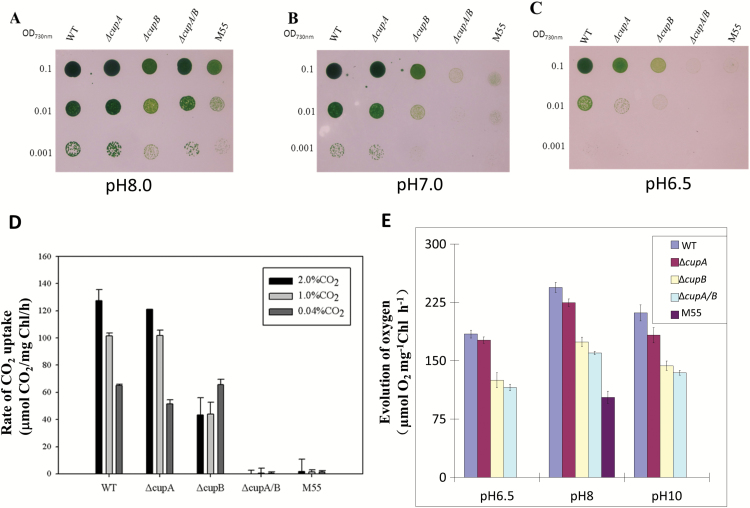
Effects of pH on the growth of wild-type and mutants on agar plates, their rates of CO_2_ uptake under various CO_2_ concentrations and their rates of photosynthetic oxygen evolution. (A–C) Five microliters of the cell suspensions with the OD_730nm_ values of 0.1, 0.01, and 0.001 were spotted on agar plates containing BG11 buffer at pH 8.0 (A), pH 7.0 (B), and pH 6.5 (C) and grown in a CO_2_ concentration of 3% for 5 days. (D) A portable photosynthesis system capable of recording the rate of CO_2_ uptake was used for measurement of CO_2_ uptake of wild-type, *ΔcupA*, *ΔcupB*, *ΔcupA/B*, and M55 under 2%, 1% and 0.04% CO_2_ concentrations on agar plates. (E) The rate of photosynthetic oxygen evolution was compared among wild type and the mutants under different pH values in the presence of 10 μM NaHCO_3_.

Figure 1A–C shows the growth of the WT, *ΔcupA* (deleted *cupA*), *ΔcupB* (deleted *cupB*), *ΔcupA/B* (both *cupA* and *cupB* deleted) and M55 (deleted *ndhB*) strains of *Synechocystis* 6803 on agar plates containing BG11 medium buffered at pH 8.0, 7.0 and 6.5, respectively, under 3% CO_2_. Five days after inoculation, there was no significant difference between the WT and *ΔcupA* strain in growth under the given conditions. The growth of *ΔcupB*, *ΔcupA/B*, and M55 mutants was slightly slower than the WT at pH 8.0. However, the growth of these mutants was severely suppressed at pH 7.0 and they hardly grew at all at pH 6.5, where Ci is predominantly, if not totally, supplied as CO_2_. The results indicate that CO_2_ is not supplied by diffusion even under 3% CO_2_ in the absence of the CO_2_-uptake systems.

Measurement of the rate of CO_2_ uptake in the WT and mutants under various CO_2_ concentrations revealed that inactivation of *cupA* had little effect on the activity, being consistent with the growth characteristics of the *ΔcupA* mutant (Fig. 1D). In contrast, inactivation of *cupB* decreased the activity to less than half that of the WT at 1% and 2% CO_2_ but had no effect at 0.04% CO_2_ (Fig. 1D). The *ΔcupA/B* and M55 mutants were unable to take up CO_2_ even at 2% CO_2_, consistent with the inability of these mutants to grow at pH 7.0. Since the expression of *cupA* is induced by low CO_2_, the growth of cells is mainly supported by CupB under high CO_2_ conditions below pH 7, where the contribution of HCO_3_^–^ transporters is limited. Taken together, these data suggest that CupB is the key component under high CO_2_ condition and the CO_2_ uptake ability of the CupB-containing complex is dependent on the *ndh* and *cupB* genes.

### Photosynthetic oxygen evolution was decreased in Cup-deletion mutants in response to different pH values

To confirm the function of both CupA and CupB, we further compared photosynthetic capacities in response to different pH values between the cells of wild type and *cup*-deletion mutants (Fig. 1E). By comparison with the value of 250 μmol O_2_ mg^–1^ Chl h^–1^ in wild type at pH 8.0, the rate of photosynthetic oxygen evolution was suppressed slightly in *ΔcupA* (92%), significantly in *ΔcupB* (71%), more evidently in *ΔcupA/B* (65%), and most severely in M55 (42%) under the same growth condition. The suppression of photosynthetic oxygen evolution was less in *ΔcupA* at pH 6.5 (96%) but more evidently at pH 10.0 (86%), severely in *ΔcupB* (68%) and in *ΔcupA/B* (63%), and almost completely in M55 at pH 6.5 or pH 10.0. The results indicate that Cup proteins as well as NdhB contribute to the photosynthetic capacity especially under changed pH conditions.

### Identification of large and small complexes containing CupB

**Fig. 2.**
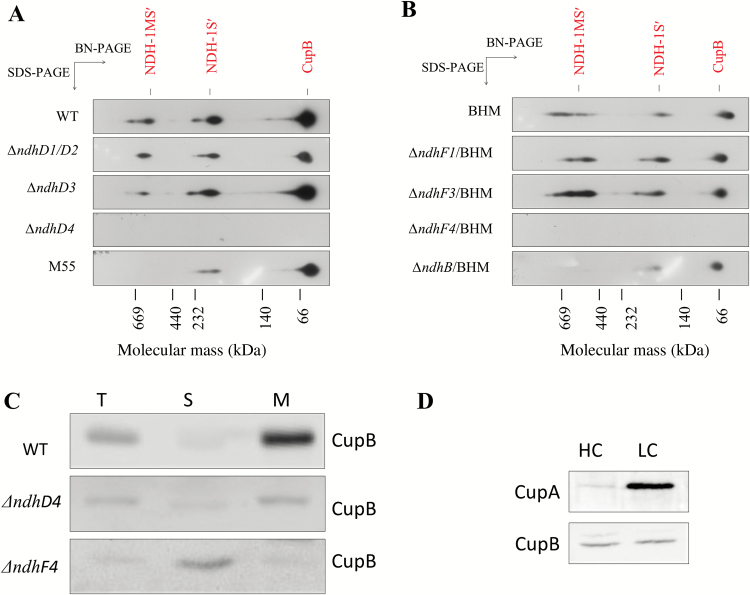
Assembly of CupB-containing complexes in different NDH-1 mutant backgrounds and localization and expression of CupB in WT, *ΔndhD4*, and *ΔndhF4.* (A) Immunodetection of CupB-containing complexes using antibody of CupB in the wild type, *ΔndhD1/D2*, *ΔndhD3*, *ΔndhD4*, *ΔndhD3/D4*, and M55 backgrounds. Total membranes complexes were separated by BN-PAGE at the first dimension and further subjected to SDS-PAGE at the second dimension. Then, immunodetections were performed with antibody of CupB. (B) Immunodetection of CupB-containing complexes in the BHM, *ΔndhF1*/BHM, *ΔndhF3*/BHM, and *ΔndhF4*/BHM backgrounds. (C) Comparison of the amount of CupB in different fractions among wild type, *ΔndhD4*, and *ΔndhF4*. The supernatant and membranes were separated and immunodetected with antibody against CupB. M, the thylakoid membrane proteins; S, the supernatant proteins; T, total proteins. (D) Accumulation of CupA and CupB in different fractions from cells of wild type grown under high CO_2_ (HC) and low CO_2_ (LC). Proteins were loaded on an equal chlorophyll basis. (This figure is available in color at *JXB* online.)

To study CupB-containing complexes in more detail, we made polyclonal CupB antibody, which cross-reacted specifically with CupB but not with CupA (see Supplementary Fig. S2). Total membrane fractions isolated from the WT and BHM cells grown under 3% CO_2_ at pH 8.0 were solubilized by *n*-dodecyl-β-D-maltoside and subjected to BN-PAGE followed by SDS-PAGE in the second dimension. Immunoblotting of the proteins electrotransferred to a PVDF membrane with antibody against CupB or α-Myc revealed three CupB-containing bands: a large band of about 500 kDa and a small one of about 200 kDa, as well as free CupB (Fig. 2A, B). The large and small bands correspond to the NDH-1MS′ and NDH-1S′ complexes, respectively.

### Assembly of CupB to NDH-1MS′ was dependent on NdhD4 and NdhF4

Genetic studies have suggested that CupB might be associated with NdhD4 and NdhF4 (Xu *et al.*, 2008). To test this possibility, thylakoid membranes of the WT and mutants were subjected to two-dimensional BN-PAGE/SDS-PAGE analysis. The immunoblot profiles of the *ΔndhD1/D2* and *ΔndhD3* mutants showed the profile of NDH-1MS′, NDH-1S′ and free CupB bands to be essentially similar to that of WT (Fig. 2A). To the contrary, none of these bands was found in the thylakoid membrane of the *ΔndhD4* mutant. Similar results were obtained with the *ndhF*-deletion mutants: deletion of *ndhF1* and *ndhF3* had no effect on the CupB-containing bands but deletion of *ndhF4* completely abolished these bands in the thylakoid membrane (Fig. 2B). It is evident that CupB is associated with NdhD4 and NdhF4 in the CupB-containing complexes. The NDH-1S′ complex is similar to NDH-1S in size and the NDH-1MS′ complex is similar to NDH-1MS in size. Analysis of the membranes and soluble fractions by western blot indicated that CupB was localized in the thylakoid membrane in the WT whereas in the *ΔndhF4* mutant it was present only in the soluble fraction (Fig. 2C), indicating that NdhF4 is essential for attachment of CupB to the thylakoid membranes. The absence of NDH-1MS′ in M55 and *ΔndhB*/BHM suggests that the assembly of NDH-1MS′ requires NdhB (Fig. 2A, B). The amount of CupA expressed was less than CupB grown under high CO_2_ conditions but was induced under low CO_2_ conditions (Fig. 2D), consistent with a previous observation of transcript levels (Shibata *et al.*, 2001).

### NDH-1MS and NDH-1MS′ independently exist

**Fig. 3.**
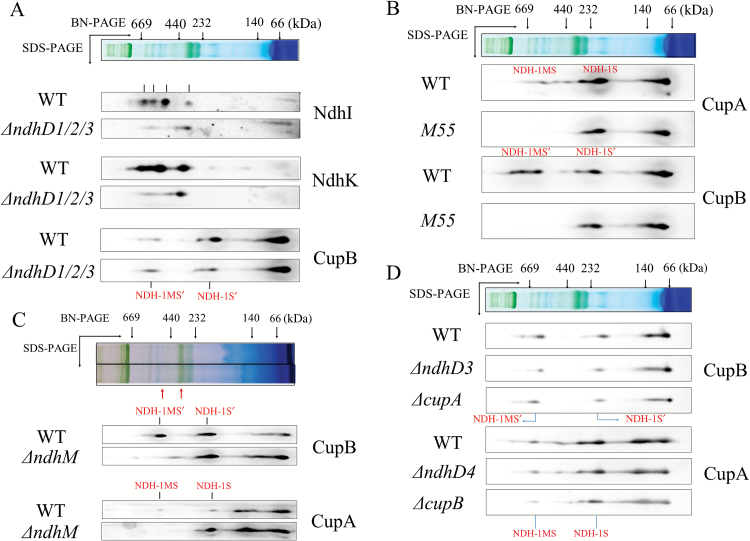
The localization of NDH-1MS and NDH-1MS′ complexes in wild type and different mutant backgrounds. The thylakoid membrane proteins from the wild type and indicated mutant strains were separated by BN-PAGE and further subjected to 2-D/SDS-PAGE. Then the proteins were immunodetected with the indicated antibodies against the Ndh subunits or Cup proteins. The co-localization of Ndh subunits and CupA in a larger molecular size band is defined as NDH-1MS while that with smaller molecular size is NDH-1S; the co-localization of Ndh subunits and CupB in the larger molecular band is NDH-1MS′ while that with smaller molecular size is NDH-1S′. (A) Comparison of accumulation of Ndh subunits, CupB and their assembly into NDH-1MS′ in wild type and *ΔndhD1/D2D3*. (B) Comparison of accumulation of CupA and CupB and their assembly into NDH-1MS and NDH-1MS′ in wild type and M55. (C) Comparison of accumulation of CupA and CupB and their assembly into NDH-1MS and NDH-1MS′ in wild type and *ΔndhM*. (D) Comparison of the accumulation of CupA and CupB and their assembly into NDH-1MS′ in wild type, *ΔcupA*, and *ΔndhD3*, and NDH-1MS in wild type, *ΔcupB*, and *ΔndhD4*. The red arrow which indicates the higher molecular site is NDH-1L and the arrow that indicates the lower one is NDH-1M. (This figure is available in color at *JXB* online.)

To distinguish NDH-1MS′ from NDH-1MS, thylakoid membranes of the WT and *ΔndhD1/D2/D3* strains were subjected to two-dimensional BN/SDS-PAGE analyses. Immunoblotting with antibodies against CupB, NdhK, and NdhI confirmed that CupB-containing complexes (NDH-1MS′ andNDH-1S′) appeared in *ΔndhD1/D2/D3* strains as well as in wild type where CupB co-localized with Ndh subunits such as NdhI and NdhK (Fig. 3A). In NdhB deletion mutant M55, both NDH-1MS and NDH-1MS′ were degraded, but NDH-1S and NDH-1S′ were still detected in *ndhB* deletion mutant M55 (Fig. 3B) and in *ndhM* deletion mutant (Fig. 3C). Furthermore, to know whether NDH-1MS and NDH-1MS′ are associated, we checked the localization of the complexes in different background mutants. As shown in Fig. 3D, detection of NDH-1MS′ and NDH-1S′ in *ΔndhD3* and *ΔcupA* was unchanged from the wild type (Zhang *et al.*, 2004). This is also true for detection of the NDH-1MS and NDH-1S complexes in *ΔndhD4* and *ΔcupB*. The NDH-1MS′ and NDH-1MS complexes were degraded in the mutants *ΔndhD4* and *ΔndhD3*, respectively (Fig. 2A). These results indicate that both NDH-1MS and NDH-1MS′ exist independently.

### Analysis of proton gradient across thylakoid membranes in the NDH-1 mutant backgrounds

**Fig. 4.**
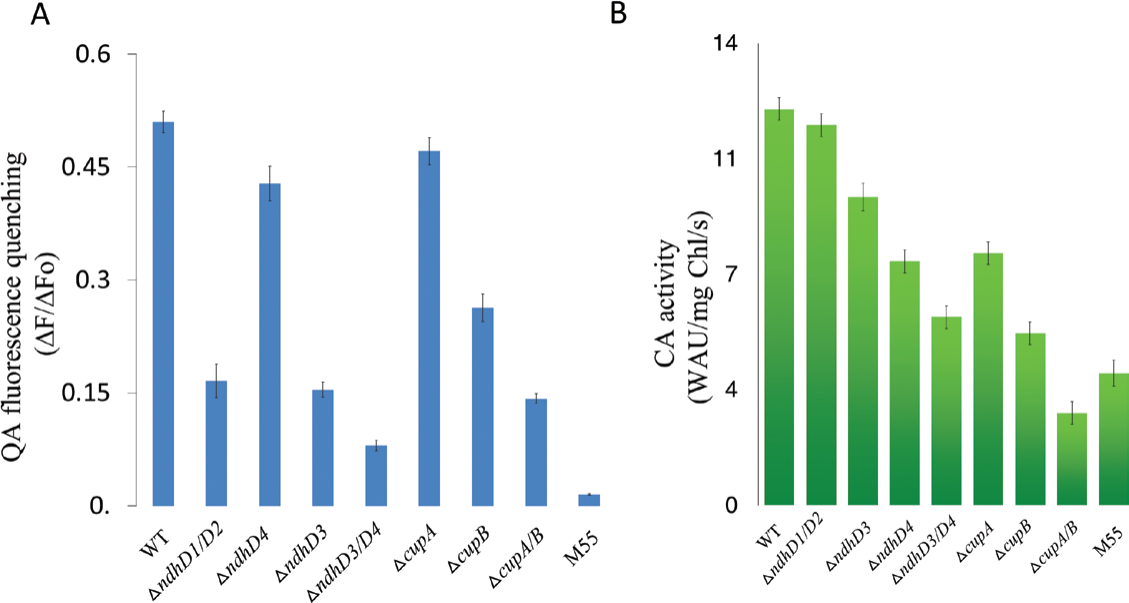
Comparison of light-induced proton gradient across thylakoid membranes and the carbonic anhydrase activities among WT, *ΔndhD1/D2*, *ΔndhD3*, *ΔndhD4*, *ΔndhD3/D4*, *ΔcupA*, *ΔcupB*, *ΔcupA/B*, and M55. (A) Intact cells of WT, *ΔndhD1/D2*, *ΔndhD3*, *ΔndhD4*, and M55 and *ΔcupA*, *ΔcupB*, and *ΔcupA/B* were harvested at midlogarithmic phase (OD_730_=0.4) and then suspended at a final chlorophyll concentration of 10 μg ml^–1^ in fresh BG11 medium with 5 μM quinacridine (QA). The quenching of QA fluorescence was induced by illumination with actinic light (60 μmol photos m^–2^ s^–1^) after starting measurement. The QA fluorescence quenching was calculated as the ratio (∆*F*/∆*F*_o_) of the decreased fluorescence intensity (∆*F*) to the background fluorescence intensity (∆*F*_o_). (B) The thylakoid membranes from these strains were suspended in 20 mM Tricine buffer at a final chlorophyll concentration of 1 mg ml^–1^. Then, samples containing 100 μg of chlorophyll were added to 8 ml 20 mM sodium barbital, pH 8.2 with a pH electrode inserted into the assay solution. After the temperature equilibrated, 4 ml ice-cold CO_2_-saturated water was injected, and the time for the pH to change from 8.0 to 7.0 was recorded. The buffer without membrane fraction was used as the control. CA activity was calculated as the difference in the initial rate of CO_2_ hydration between the control and the samples. Values are the averages of four independent measurements. Standard errors are indicated by the vertical bars. (This figure is available in color at *JXB* online.)

It has been suggested that cyanobacterial NDH-1 provides ATP for CO_2_ uptake (Ogawa, 1991). To confirm whether the NDH-1 complex contributes to the proton gradient across thylakoid membranes, a driving force for synthesis of ATP, light-induced quenching of quinacridine (QA) fluorescence for the determination of ΔpH across the thylakoid membrane for intact cells of *Synechocystis* 6803 (Teuber *et al.*, 2001) was measured in different NDH-1 mutants backgrounds. As shown in Fig. 4A, the quenching of QA fluorescence was remarkably suppressed in M55 (by 97%), *ΔndhD3/4* (by 84%), *ΔcupA/B* (by 72%), *ΔndhD4* (by 69%), *ΔndhD1/D2* (by 67%), *ΔcupB* (by 49%), partly in *ΔndhD3* (by 16%) and slightly in *ΔcupA* (by 8%) compared with wild type. Those results suggested that both NDH and Cup proteins are involved in building up the proton gradient across thylakoid membrane and NdhD4 is a key component in this process.

### Carbonic anhydrase activity was suppressed in the NDH- and Cup-deletion mutants

To further investigate the mechanism of NDH-1MS and NDH-1MS′ in the conversion of CO_2_ to HCO_3_^–^, we measured the CA activity of the membrane proteins isolated from the wild type, *ΔndhD1/D2*, *ΔndhD3*, *ΔndhD4*, *ΔndhD3/D4*, *ΔcupA*, *ΔcupB*, *ΔcupA/B*, and M55 (Fig. 4B). Compared with wild type, CA activity was most greatly lowered in both *ΔcupA/B* (23%) and M55 (33%), more significantly suppressed in *ΔcupB* (43%), and *ΔndhD3/D4* (47%), slightly in *ΔndhD4* (62%), *ΔcupA* (63%) and *ΔndhD3* (78%) compared with wild type, but almost not affected in *ΔndhD1/D2*. Those results demonstrate that the Cup proteins are involved in the conversion of CO_2_ to HCO_3_^–^ as CA-like proteins.

## Discussion

### NdhD4 and NdhF4 are essential for the assembly of CupB-containing complexes

Although NDH-1MS′ was identified, its assembly is still unclear. In this work, we show evidence that NdhD4 and NdhF4 are crucial for the assembly of NDH-1MS′, based on the result that when *ndhF4* was knocked out, the CupB was completely missing from the thylakoid membrane, but was found soluble in the cytoplasm (Fig. 2C), probably resulting in no detection of NDH-1MS′ or NDH-1S′ (Fig. 2B). On the other hand, when *ndhD4* was deleted, this significantly decreased the accumulation of CupB in the thylakoid membrane (Fig. 2C), as reported in our previous study (Xu *et al.*, 2008), and also resulted in degradation of NDH-1MS′ or NDH-1S′ (Fig. 2A). We further show that NdhD4 is co-located with CupB, evidenced by detecting the strep tag fused to the C-terminus of NdhD4 (see Supplementary Figs S3 and S4A). The recovery of the assembly of CupB-containing complexes by complementing *ndhF4* gene to *ΔndhF4* mutant (Supplementary Figs S3 and S4B) provided further evidence for the crucial role of NdhF4 in the NDH-1S′ complex. Recently an NDH-1S′ complex containing NdhD4, NdhF4, and CupB has been isolated from a *Thermosynechoccus elongatus* with twin-strep tagged to NdhL (Wulfhorst *et al.*, 2014). We also found that when *ndhB* is deleted, NDH-1S′ can still be normally assembled (Figs 2A and 3B), but the ability to take up CO_2_ is severely compromised (Fig. 1D), indicating that NDH-1S′ alone is not functional in CO_2_ uptake.

### Co-ordination of NdhDs and Cup proteins in CO_2_ uptake activity

**Fig. 5.**
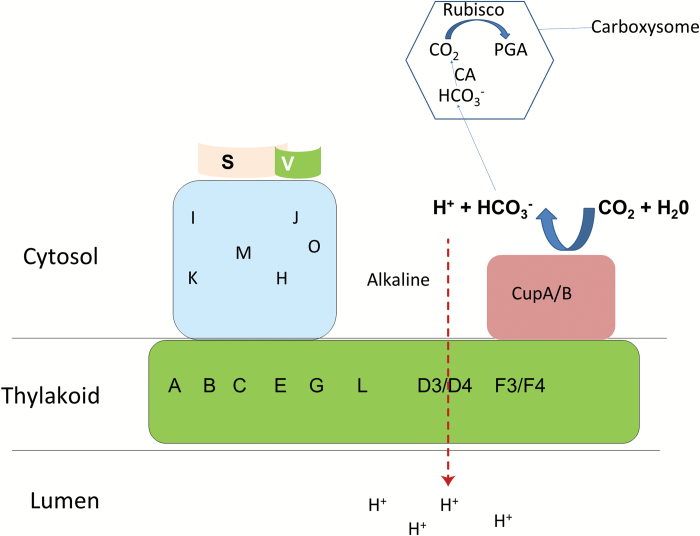
A model of the proposed function of CO_2_ uptake systems in *Synechocystis* sp. strain PCC6803. CupA or CupB converts CO_2_ into HCO_3_^–^ under alkaline conditions, while the conversion is reversed under acidic conditions. Under light conditions, photosynthetic electron transfer couples to the formation of a transthylakoid membrane proton gradient, subsequently forming a strong alkaline region inside the U-type structure for CupA or CupB activity, which leads to the accumulation of HCO_3_^–^ after CO_2_ has diffused into the cytosol. After HCO_3_^–^ enters the carboxysome, it is converted into CO_2_ by CA for carbon assimilation by Rubisco. (This figure is available in color at *JXB* online.)

By resolution of mitochondrial complex I structure, it has been suggested that complex I regulates the transmembrane proton gradient by its conformation change during electron transfer (Royer *et al.*, 2006; Efremov *et al.*, 2010; Vinothkumar *et al.*, 2014). The similar function has been proposed in NDH-1 previously (Battchikova *et al.*, 2011). In this work, we further found that the proton gradient across the thylakoid membrane was significantly suppressed in *ΔndhD4* and even more in *ΔndhD3/D4*, *ΔndhD1/D2*, and M55 (Fig. 4A), suggesting NdhD subunits, mainly NdhD4, function as a proton pump to provide a proton gradient across the thylakoid membranes, as it is homologous to subunit M in complex I, which drives the ATPase, to synthesize ATP for active CO_2_ uptake and for regulation of pH in the cytosol. The lesser contribution of NdhD3 to proton pumping than NdhD4 might be attributable to its lesser expression (Ohkawa *et al.*, 1998) and lesser amount (Fig. 2D) under the high CO_2_ culture condition. The partial suppression of QA fluorescence in *ΔcupB* suggests that CupB is also involved in building up a sufficient proton gradient across the thylakoid membrane (Fig. 4A). Although the formation of the transthylakoid membrane ∆pH was only slightly affected in *ΔcupA*, it was significantly decreased in *ΔcupA/B* (Fig. 4A), which led us to conclude that the formation of ∆pH by NDH-1MS and NDH-1MS′ complexes requires the coordination of Cup proteins for providing protons in the hydration of CO_2_ to HCO_3_^–^ (Fig. 5).

### The possible role of CupA/CupB and NDH-1M in regulation of CO_2_ uptake

There are four kinds of carbonic anhydrase forming a small complex located in Complex I of Arabidopsis that are suggested to be crucial for the balance of CO_2_ and HCO_3_^–^ (Meyer *et al.*, 2011; Li *et al.*, 2013; Wydro *et al.*, 2013). Kaplan *et al* also suggested that a similar structure of the carbonic anhydrase might exist in cyanobacteria (Kaplan and Reinhold, 1999). The low carbonic anhydrase activity in *ΔcupA/B* (Fig. 4B) indicates that Cup proteins, mainly CupB, function as a carbonic anhydrase to convert CO_2_ into HCO_3_^–^. On the other hand, in the NDH-1MS′ degradation mutants, including *ΔndhD4*, *ΔndhD3/D4*, and M55 (Fig. 2), not only the building up of a transthylakoid membrane proton gradient (Fig. 4A) but also the activity of carbonic anhydrase (Fig. 4B) was suppressed. This allows us to conclude that Ndh subunits are required for the activation of the carbonic anhydrase. The function of carbonic anhydrase depends on the environment. Carbonic anhydrase converts CO_2_ into HCO_3_^–^ under alkaline conditions while the conversion is reversed under acidic conditions (Kupriyanova and Pronina, 2011). CupA or CupB might have a similar function as they also display CA activity (Fig. 4B). In cyanobacterial cytosol, the inorganic carbon source exists as HCO_3_^–^ whose accumulation might require an alkaline environment. Based on our results in Fig. 4, we suggest that the transthylakoid membrane proton gradient through NdhDs coupled with the cyclic electron flow around PS I mediated by NDH-1M (He *et al.*, 2016; He and Mi, 2016) might create a strong alkaline region in the cytosol (lumen becomes more acidic and cytosol become more alkaline) as suggested previously (Kaplan and Reinhold, 1999), suitable for Cup proteins to hydrate CO_2_ into HCO_3_^–^ in the cytosol (Fig. 5).

### The relationship of CupB and CupA in the CO_2_ uptake pathway

CupB is a homologous protein of CupA with 40% similarity. Xu *et al*. (2008) found that the expression of CupB is constitutive and not affected by CO_2_ concentration, while the expression of CupA is induced by low CO_2_ and is involved in CO_2_ uptake under low CO_2_ conditions. In this work, we found that in addition to the main function of CupB, CupA also functioned at high CO_2_ conditions (Figs 1 and 2), in accordance with the results of the isolated NDH-1S from high CO_2_-cultured cells (Wulfhorst *et al.*, 2014), suggesting CupA was also expressed under high CO_2_ conditions. The mutant defective in both CupA and CupB hardly grew on the agar plate at pH lower than 7.0 even at 3% CO_2_ (Fig. 1B), and the photosynthetic oxygen evolution was also evidently suppressed (Fig. 1E), suggesting that diffusion of CO_2_ through cells to the carboxylation site is insignificant in the absence of CO_2_-uptake systems, and that both CupA and CupB are required for efficient CO_2_ uptake (Fig. 1C).

In conclusion, using reverse genetics and biochemical methods, we investigated the function of NDH-1MS′ and NDH-1MS in CO_2_ uptake. Based on our results, we propose that the transthylakoid membrane proton gradient coupled by the electron transport mediated by NDH-1M might create an alkaline region, suitable for CupA or CupB to convert CO_2_ into HCO_3_^–^ in the cytosol (Fig. 5). However, further experimental data to support this hypothesis are still needed.

## Supplementary data

Supplementary data are available at *JXB* online.

Fig. S1. Construction and segregation of *ndhF* and *ndhD* mutants.

Fig. S2. Polyclonal antibody of CupB preparation and immunological characterization of CupB in mutants.

Fig. S3. Construction and segregation check of *ndhD4HA*/BHM, *ndhD4HA*/*ΔndhF4*/BHM, *ndhF4HA*/BHM, and *ndhF4HA*/*ΔndhF4*/BHM, and *ndhD4*strep.

Fig. S4. Association of CupB with NdhD4 and NdhF4.

Table S1. Primer details used in construction and isolation of mutants.

## Supplementary Material

Supplementary_Table_S1_Figures_S1_S4

## Abbreviations:

CCM: CO_2_-concentrating mechanism
CET: cyclic electron flow
NDH: NAD(P)H dehydrogenase
PQ: plastoquinone
PS: photosystem.

## References

[CIT0001] Arteni AA Zhang P Battchikova N Ogawa T Aro EM Boekema EJ 2006 Structural characterization of NDH-1 complexes of *Thermosynechococcus elongatus* by single particle electron microscopy. Biochimica et Biophysica Acta 1757, 1469–1475. 16844076 10.1016/j.bbabio.2006.05.042

[CIT0002] Battchikova N Eisenhut M Aro EM 2011 Cyanobacterial NDH-1 complexes: novel insights and remaining puzzles. Biochimica et Biophysica Acta 1807, 935–944. 21035426 10.1016/j.bbabio.2010.10.017

[CIT0003] Birungi M Folea M Battchikova N Xu M Mi H Ogawa T Aro EM Boekema EJ 2010 Possibilities of subunit localization with fluorescent protein tags and electron microscopy examplified by a cyanobacterial NDH-1 study. Biochimica et Biophysica Acta 1797, 1681–1686. 20547137 10.1016/j.bbabio.2010.06.004

[CIT0004] Chen X He Z Xu M Peng L Mi H 2016 NdhV subunit regulates the activity of type-1 NAD(P)H dehydrogenase under high light conditions in cyanobacterium *Synechocystis* sp. PCC 6803. Scientific Reports 6, 28361–28361. 27329499 10.1038/srep28361 PMC4916593

[CIT0005] Efremov RG Baradaran R Sazanov LA 2010 The architecture of respiratory complex I. Nature 465, 441–445. 20505720 10.1038/nature09066

[CIT0006] Folea IM Zhang P Nowaczyk MM Ogawa T Aro EM Boekema EJ 2008 Single particle analysis of thylakoid proteins from *Thermosynechococcus elongatus* and *Synechocystis* 6803: localization of the CupA subunit of NDH-1. FEBS Letters 582, 249–254. 18083126 10.1016/j.febslet.2007.12.012

[CIT0007] He Z Mi H 2016 Functional characterization of the subunits N, H, J, and O of the NAD(P)H dehydrogenase complexes in *Synechocystis* sp. strain PCC 6803. Plant Physiology 171, 1320–1332. 27208236 10.1104/pp.16.00458 PMC4902626

[CIT0008] He Z Xu M Wu Y Lv J Fu P Mi H 2016 NdhM subunit is required for the stability and the function of NAD(P)H dehydrogenase complexes involved in CO_2_ uptake in *Synechocystis* sp. strain PCC 6803. The Journal of Biological Chemistry 291, 5902–5912. 26703473 10.1074/jbc.M115.698084 PMC4786724

[CIT0009] Hu P Lv J Fu P Hualing M 2013 Enzymatic characterization of an active NDH complex from *Thermosynechococcus elongatus*. FEBS Letters 587, 2340–2345. 23722112 10.1016/j.febslet.2013.05.040

[CIT0010] Jiang HB Cheng HM Gao KS Qiu BS 2013 Inactivation of Ca^2+^/H^+^ exchanger in *Synechocystis* sp. strain PCC 6803 promotes cyanobacterial calcification by upregulating CO_2_-concentrating mechanisms. Applied and Environmental Microbiology 79, 4048–4055. 23624472 10.1128/AEM.00681-13 PMC3697565

[CIT0011] Kaplan A Reinhold L 1999 CO_2_ concentrating mechanisms in photosynthetic microorganisms. Annual Review of Plant Physiology and Plant Molecular Biology 50, 539–570. 10.1146/annurev.arplant.50.1.539 15012219

[CIT0012] Kupriyanova EV Pronina NA 2011 Carbonic anhydrase: enzyme that has transformed the biosphere. Russian Journal of Plant Physiology 58, 197–209.

[CIT0013] Laemmli UK 1970 Cleavage of structural proteins during the assembly of the head of bacteriophage T4. Nature 227, 680–685. 5432063 10.1038/227680a0

[CIT0014] Li L Nelson CJ Carrie C Gawryluk RM Solheim C Gray MW Whelan J Millar AH 2013 Subcomplexes of ancestral respiratory complex I subunits rapidly turn over *in vivo* as productive assembly intermediates in *Arabidopsis*. The Journal of Biological Chemistry 288, 5707–5717. 23271729 10.1074/jbc.M112.432070 PMC3581425

[CIT0015] Maeda S Badger MR Price GD 2002 Novel gene products associated with NdhD3/D4-containing NDH-1 complexes are involved in photosynthetic CO_2_ hydration in the cyanobacterium, *Synechococcus* sp. PCC7942. Molecular Microbiology 43, 425–435. 11985719 10.1046/j.1365-2958.2002.02753.x

[CIT0016] Meyer EH Solheim C Tanz SK Bonnard G Millar AH 2011 Insights into the composition and assembly of the membrane arm of plant complex I through analysis of subcomplexes in *Arabidopsis* mutant lines. The Journal of Biological Chemistry 286, 26081–26092. 21606486 10.1074/jbc.M110.209601 PMC3138247

[CIT0017] Ogawa T 1991 A gene homologous to the subunit-2 gene of NADH dehydrogenase is essential to inorganic carbon transport of *Synechocystis* PCC6803. Proceedings of the National Academy of Sciences, USA 88, 4275–4279. 10.1073/pnas.88.10.4275 PMC51641 1903537

[CIT0018] Ogawa T Kaplan A 2003 Inorganic carbon acquisition systems in cyanobacteria. Photosynthesis Research 77, 105–115. 16228369 10.1023/A:1025865500026

[CIT0019] Ogawa T Mi H 2007 Cyanobacterial NADPH dehydrogenase complexes. Photosynthesis Research 93, 69–77. 17279442 10.1007/s11120-006-9128-y

[CIT0020] Ohkawa H Pakrasi HB Ogawa T 2000a Two types of functionally distinct NAD(P)H dehydrogenases in *Synechocystis* sp. strain PCC6803. The Journal of Biological Chemistry 275, 31630–31634. 10906128 10.1074/jbc.M003706200

[CIT0021] Ohkawa H Price GD Badger MR Ogawa T 2000b Mutation of *ndh* genes leads to inhibition of CO_2_ uptake rather than HCO_3_^–^ uptake in *Synechocystis* sp. strain PCC 6803. Journal of Bacteriology 182, 2591–2596. 10762263 10.1128/jb.182.9.2591-2596.2000 PMC111325

[CIT0022] Ohkawa H Sonoda M Katoh H Ogawa T 1998 The use of mutants in the analysis of the CO_2_-concentrating mechanism in cyanobacteria. Canadian Journal of Botany–Revue Canadienne De Botanique 76, 1035–1042.

[CIT0023] Price GD 2011 Inorganic carbon transporters of the cyanobacterial CO_2_ concentrating mechanism. Photosynthesis Research 109, 47–57. 21359551 10.1007/s11120-010-9608-y

[CIT0024] Royer WE Jr Sharma H Strand K Knapp JE Bhyravbhatla B 2006 *Lumbricus* erythrocruorin at 3.5 angstrom resolution: architecture of a megadalton respiratory complex. Structure 14, 1167–1177. 16843898 10.1016/j.str.2006.05.011

[CIT0025] Shibata M Ohkawa H Kaneko T Fukuzawa H Tabata S Kaplan A Ogawa T 2001 Distinct constitutive and low-CO_2_-induced CO_2_ uptake systems in cyanobacteria: Genes involved and their phylogenetic relationship with homologous genes in other organisms. Proceedings of the National Academy of Sciences, USA 98, 11789–11794. 10.1073/pnas.191258298 PMC58809 11562454

[CIT0026] Teuber M Rögner M Berry S 2001 Fluorescent probes for non-invasive bioenergetic studies of whole cyanobacterial cells. Biochimica et Biophysica Acta 1506, 31–46. 11418095 10.1016/s0005-2728(01)00178-5

[CIT0027] Vinothkumar KR Zhu J Hirst J 2014 Architecture of mammalian respiratory complex I. Nature 515, 80–84. 25209663 10.1038/nature13686 PMC4224586

[CIT0028] Wulfhorst H Franken LE Wessinghage T Boekema EJ Nowaczyk MM 2014 The 5 kDa protein NdhP is essential for stable NDH-1L assembly in *Thermosynechococcus elongatus*. PLoS ONE 9, e103584. 25119998 10.1371/journal.pone.0103584 PMC4131877

[CIT0029] Wydro MM Sharma P Foster JM Bych K Meyer EH Balk J 2013 The evolutionarily conserved iron-sulfur protein INDH is required for complex I assembly and mitochondrial translation in *Arabidopsis*. The Plant Cell 25, 4014–4027. 24179128 10.1105/tpc.113.117283 PMC3877808

[CIT0030] Xu M Ogawa T Pakrasi HB Mi H 2008 Identification and localization of the CupB protein involved in constitutive CO_2_ uptake in the cyanobacterium, *Synechocystis* sp. strain PCC 6803. Plant & Cell Physiology 49, 994–997. 18467341 10.1093/pcp/pcn074

[CIT0031] Xu M Shi N Li Q Mi H 2014 An active supercomplex of NADPH dehydrogenase mediated cyclic electron flow around Photosystem I from the panicle chloroplast of *Oryza sativa*. Acta Biochimica et Biophysica Sinica 46, 757–765. 25074414 10.1093/abbs/gmu064

[CIT0032] Zhang P Battchikova N Jansen T Appel J Ogawa T Aro EM 2004 Expression and functional roles of the two distinct NDH-1 complexes and the carbon acquisition complex NdhD3/NdhF3/CupA/Sll1735 in *Synechocystis* sp PCC 6803. The Plant Cell 16, 3326–3340. 15548742 10.1105/tpc.104.026526 PMC535876

[CIT0033] Zhang P Battchikova N Paakkarinen V Katoh H Iwai M Ikeuchi M Pakrasi HB Ogawa T Aro EM 2005 Isolation, subunit composition and interaction of the NDH-1 complexes from *Thermosynechococcus elongatus* BP-1. The Biochemical Journal 390, 513–520. 15910282 10.1042/BJ20050390 PMC1198931

