# Supplementary material for: Co-ordination of NDH and Cup proteins in CO2 uptake in cyanobacterium Synechocystis sp. PCC 6803
Source: J Exp Bot. 2017 May 10;68(14):3869–77. doi: 10.1093/jxb/erx129 (PMC5853218; doi:10.1093/jxb/erx129)
Supplement: Supplementary_Table_S1_Figures_S1_S4 [file erx129_suppl_supplementary_table_s1_figures_s1_s4.pdf]

| | |
|---|---|
| hxl108 | 5’-ATTGTTGACGTTCCACCAGGGTTA-3’ |
| hxl109 | 5’-TGAAAATCTCGCCAAGCTTGTCAAGCCAGGCTAATTGATAGAGTAA-3’ |
| hxl110 | 5’-TTACTCTATCAATTAGCCTGGCTTGACAAGCTTGGCGAGATTTTCA-3’ |
| hxl111 | 5’-CAAATAGGGGCAGGACGGGAATTCAGGCGTAGCAACCAGGCGTTTA-3’ |
| hxl112 | 5’-TAAACGCCTGGTTGCTACGCCTGAATTCCCGTCCTGCCCCTATTTG-3’ |
| hxl113 | 5’-CCCCGTCTTTCGCTTCCTGGTCATT-3’ |
| hxl114 | 5’-ACTAATTGTCATGCAGGGTTCTCC-3’ |
| hxl115 | 5’-CTGAAAATCTCGCCAAGCTTGTCACTAACATGGGGGTTGTGGGAAATT-3’ |
| hxl116 | 5’-AATTTCCCACAACCCCCATGTTAGTGACAAGCTTGGCGAGATTTTCAG-3’ |
| hxl117 | 5’-GCCAGATAATTCGACTTAAACTTTCAGGCGTAGCAACCAGGCGTTTAA-3’ |
| hxl118 | 5’-TTAAACGCCTGGTTGCTACGCCTGAAAGTTTAAGTCGAATTATCTGGC-3’ |
| hxl119 | 5’-GAGCAAAACCTACTGTCACAACCAT-3’ |

**Table S1 Primer Details Used in Construction and isolation of Mutants**

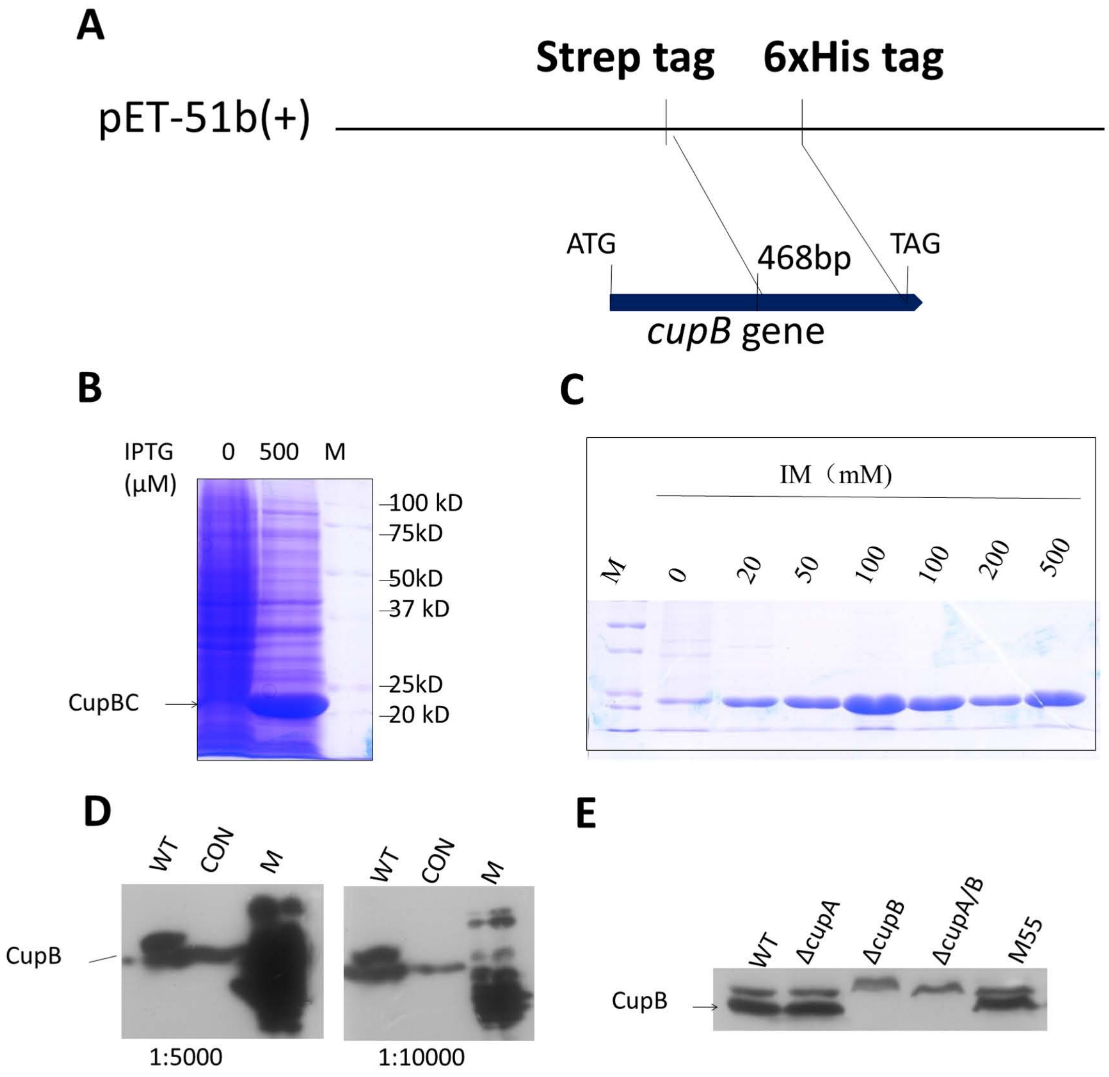


**FIG.S1 Polyclonal antibody of CupB preparation and immunological characterization of CupB in mutants.**

A. Construction of expression vector of CupB C-terminal (CupBC). B. expression analysis of CupB C-terminal. CupBC: CupB C-terminal , M: protein marker. C. purification of CupB C-terminal with $Ni^{2+}$-NTA-agarose resin. IM: imidazole. D. immunological characterization of CupB. CON: CupB-His-Myc. M: protein marker. E. immunological characterization of CupB in different mutants.

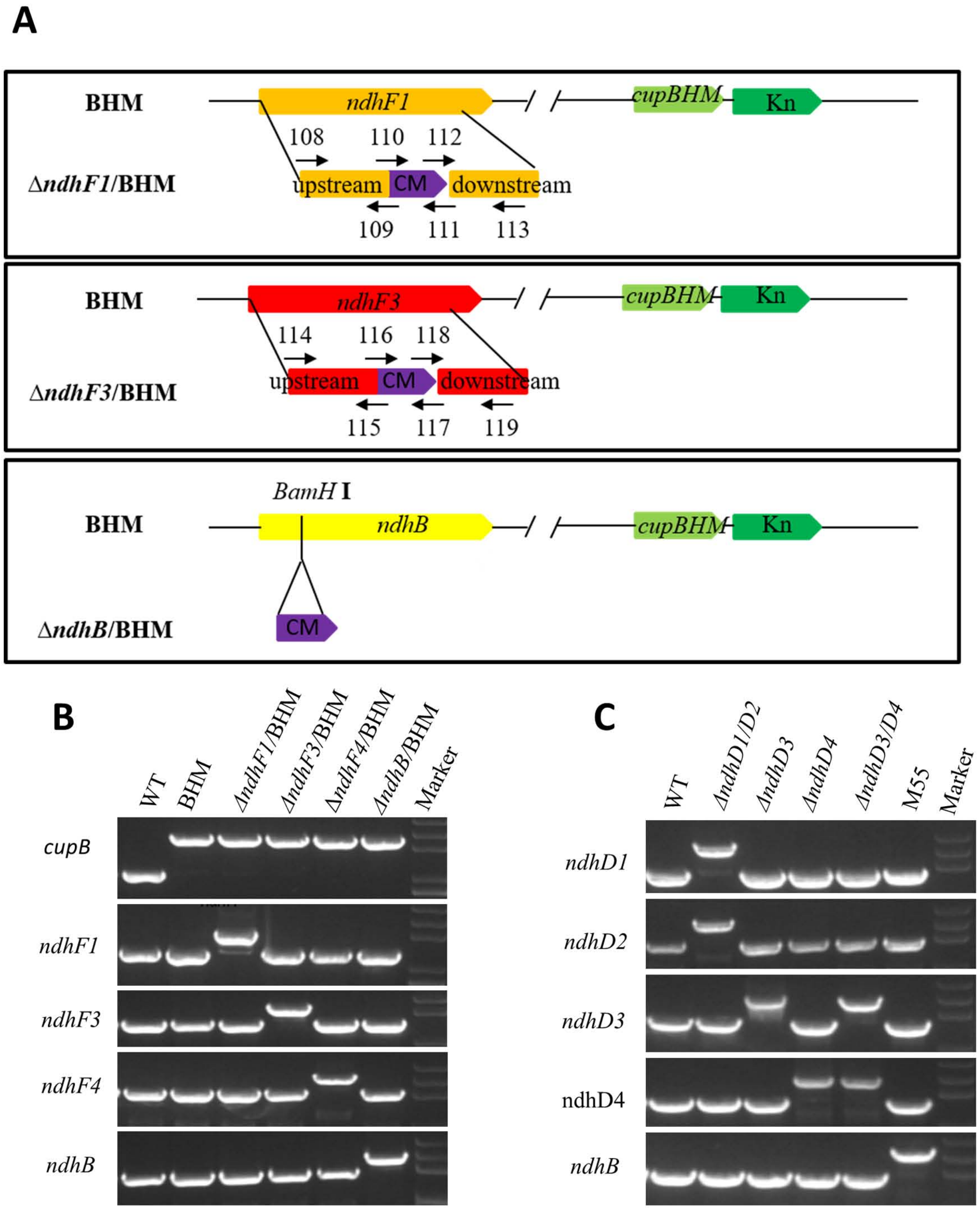


**FIG.S2 Construction and segregation of ndhF and ndhD mutants.**

**A.** chloramphenicol resistance cassette was inserted into *ndhF1*, *ndhF3* and *ndhB, separately. cupBHM*: CupB tagged with 6xHis-cMyc at the C-terminal. CM: chloramphenicol resistance cassette.

Kn: Kanamycin resistance cassette.

108-119: hxl108-hxl119, see supplemental data table 1.

**B.** The mutated genes in the transformants of ndhB and ndhF mutants were segregated to homogeneity as determined by PCR amplification. **C.** The mutated genes in the transformants of M55 and ndhDs were segregated to homogeneity as determined by PCR amplification.

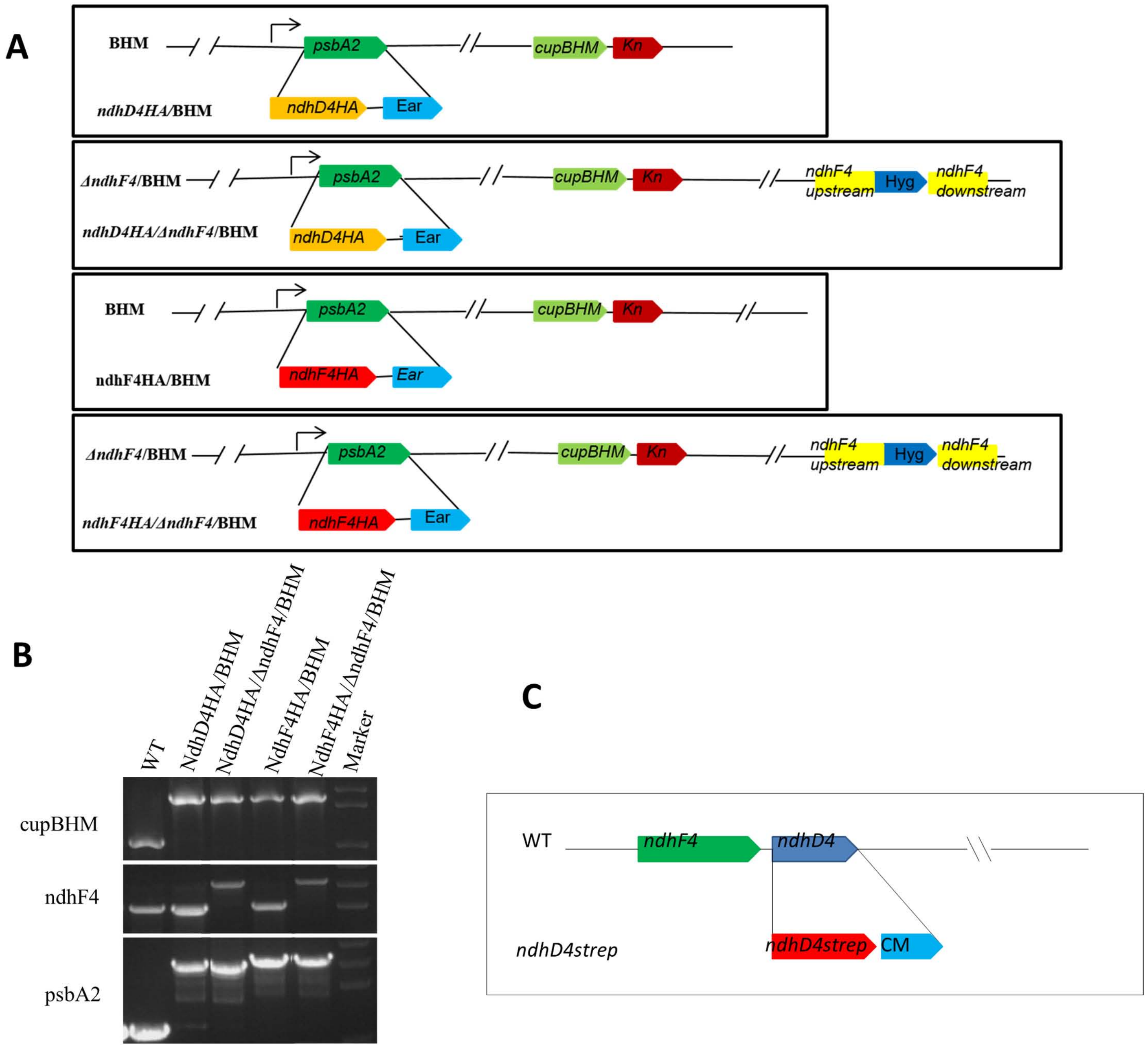
A
BHM
psbA2
cupBHM
Kn
ndhD4HA/BHM
ndhD4HA
Ear
ΔndhF4/BHM
ndhF4 upstream
Hyg
ndhF4 downstream
ndhD4HA/ΔndhF4/BHM
ndhF4HA/BHM
ndhF4HA
ndhF4HA/ΔndhF4/BHM
B
WT
NdhD4HA/BHM
NdhD4HA/ΔndhF4/BHM
NdhF4HA/BHM
NdhF4HA/ΔndhF4/BHM
Marker
cupBHM
ndhF4
psbA2
C
WT
ndhF4
ndhD4
ndhD4strep
CM

**FIG.S3 construction and segregation check of *ndhD4HA*/BHM, *ndhD4HA*/Δ*ndhF4*/BHM, *ndhF4HA*/BHM, and *ndhF4HA*/Δ*ndhF4*/BHM, and *ndhD4strep*.**

**A.** *ndhD4HA*/BHM, *ndhD4HA*/Δ*ndhF4*/BHM, *ndhF4HA*/BHM, and *ndhF4HA*/Δ*ndhF4*/BHM were constructed as shown in **FIG.S3A**. PS II A2 gene was exchanged by *ndhD4* and *ndhF4* tagged with HA gene. **B**. The mutated genes in the transformants of *ndhD4HA*/BHM, *ndhD4HA*/Δ*ndhF4*/BHM, *ndhF4HA*/BHM, and *ndhF4HA*/Δ*ndhF4*/BHM mutants were segregated to homogeneity as determined by PCR amplification. **C**. *ndhD4strep* was constructed as shown in **FIG.S3C**. *ndhD4* gene was exchanged by *ndhD4* tagged with strep gene.

psbA2: photosystem II A2 gene.

*cupBHM*: CupB tagged with 6xHis-cMyc at the C-terminal.

CM: Chloramphenicol resistance cassette.

Kn: Kanamycin resistance cassette.

Ear: Erythromycin resistance cassette.

Hyg: Hygromycin resistance cassette.

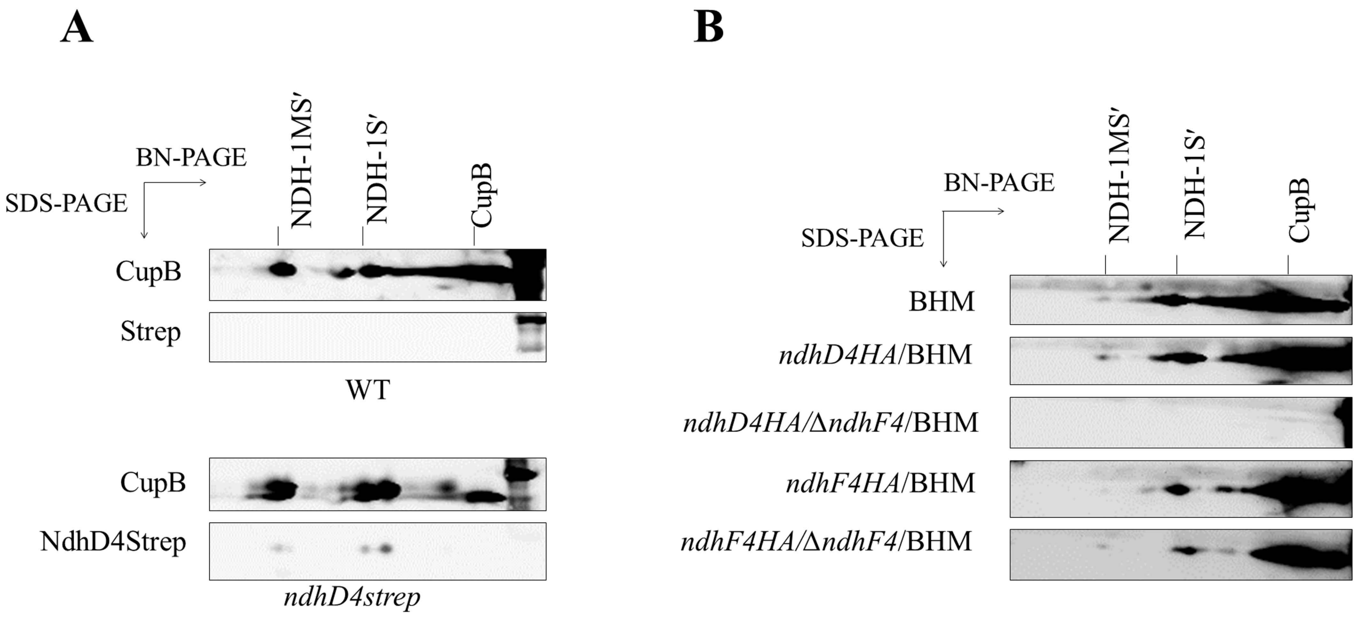


**FIG. S4.** Association of CupB with NdhD4 and NdhF4.
(A) The position of CupB and NdhD4 were detected by specific antibody. Total membrane proteins from wild type and *ndhD4strep* were separated and immunodetected with antibody against CupB and Strep, respectively. (B) Assembly of CupB complex in transformed mutants background. Membrane proteins from BHM, *ndhD4HA*/BHM, *ndhD4HA*/Δ*ndhF4*/BHM, *ndhF4HA*/BHM, and *ndhF4HA*/Δ*ndhF4*/BHM were separated by BN-SDS-PAGE, and were immunodetected with antibody against CupB.
